# Validating a short-form strategy inventory for English as a foreign language learners: testing Oxford's strategy role flexibility theory

**DOI:** 10.3389/fpsyg.2026.1863208

**Published:** 2026-07-22

**Authors:** Tsung-Yuan Hsiao, Wen-Ta Tseng

**Affiliations:** 1 Institute of Applied English, National Taiwan Ocean University, Keelung, Taiwan; 2 Department of Applied Foreign Languages, National Taiwan University of Science and Technology, Taipei City, Taiwan

**Keywords:** confirmatory factor analysis, English as a foreign language, language learning strategies, second/foreign language, strategy role flexibility

## Abstract

This study primarily validated the *SILL*-*ELL* Student Form for college EFL learners and, as a secondary aim, examined whether selected cross-loadings were consistent with Oxford's strategy role flexibility theory. Data from 509 Taiwanese college EFL learners were analyzed using theory-guided CFA, calibration–holdout cross-validation, reliability and validity analyses, and gender invariance testing. The initial six-factor simple-structure model showed inadequate fit, but a revised six-factor model fit acceptably after three items were removed, one item was reclassified, and four compensation-related cross-loadings were estimated. The resulting 25-item *SILL*-*SEFL* differed from the *SILL*-*ELL* in target population, item composition, one item classification, and the inclusion of theory-guided secondary loadings. The findings provide qualified support for Oxford's six broad strategy categories and indirect psychometric evidence consistent with strategy role flexibility; however, cross-loadings should not be interpreted as direct proof of flexible strategy use. Results support cautious use of the *SILL*-*SEFL* in comparable college EFL contexts and highlight the need for external replication.

## 1 Introduction

Since Rubin's (1975) pioneering work on “the good language learner,” research on language learning strategies has developed into a sustained line of inquiry and continues to attract renewed scholarly attention in contemporary applied linguistics (Chen and Alibakhshi, 2026; Hajar and Karakus, 2025; Lan, 2025). Researchers have examined both the strategies themselves and the factors associated with their adoption (e.g., Dang, 2024; Feng, 2025; Hsu et al., 2024; Ma et al., 2024). The scholarly interest of Rubin in “the good language learner” emerges from the realization that the strategies that characterize skilled language learners (and the strategies they employ) can provide insights into assisting those who possess lower proficiency. During subsequent efforts to define and classify these techniques of effective language learners, researchers mostly approached strategy as a single-function construct. They assigned one strategy to only one category (e.g., O'Malley and Chamot, 1990; Rubin, 1981; Saks and Leijen, 2018; for an early review, see Oxford and Burry-Stock, 1995). However, this manner of researching language learning strategies has resulted in findings that some critics describe as vague and ambiguous (e.g., Dörnyei, 2005; Dörnyei and Ryan, 2015; Rose, 2012; Tseng et al., 2006). In response to this concern, Oxford (2017) argues that a binary, overly simplistic categories approach is not only impractical but also unrealistic. She points to the multifaceted and complex nature of this area of inquiry and states that “strategy categories are not rigid but are instead flexible and permeable” (p. 141). Disagreement over classification is almost always unavoidable, as Oxford noted in the 1990s. Indeed, subsequent studies (e.g., Hsiao and Oxford, 2002; Papadopoulou et al., 2018; Petrogiannis and Gavriilidou, 2015; Robson and Midorikawa, 2001) have supported this perspective by employing the *Strategy Inventory for Language Learning* (*SILL*) of Oxford (1990).

Oxford (2017) encourages scholars to explore language learning strategies in creative ways and advocates for a more dynamic viewpoint. She stresses the significance of acknowledging the fluidity and adaptability of strategies in different contexts and functions. This perspective allows strategies to adopt various roles that align with real-world situations and objectives. From a psychometric perspective, this adaptability permits a single strategy to be included in more than one category, which can be conceptualized as a strategy “cross-loading” on multiple factors (e.g., Asparouhov and Muthén, 2009; Asparouhov et al., 2015; Brown, 2015; Byrne, 2006; Hsu et al., 2014; McNeish and Wolf, 2023; Morin et al., 2016). For example, one item from the *SILL* (Oxford, 1990) reads: “I ask English speakers to correct me when I talk.” Although it is theoretically categorized as a social strategy, it can also function as a compensation strategy because learners employ it to address limited language proficiency. The flexibility proposal of Oxford regarding strategy classification challenges many empirical findings that have traditionally sought to identify fixed strategies for language learners. Rather than treating strategies as possessing a unitary role, it is crucial to identify strategies that demonstrate flexibility and can serve multiple roles. When these strategies are recognized, learners can develop a more effective approach to strategy use and self-regulated learning, since they can modify their strategies for various contexts and purposes.

Although Oxford's (2017) strategy role flexibility theory is theoretically promising, its measurement implications require further empirical examination. The primary purpose of the present study was therefore to cross-culturally validate Ardasheva and Tretter's (2013) *SILL*-*ELL* (*Strategy Inventory for Language Learning*—*English Language Learner*) Student Form with college EFL learners in Taiwan. Because the *SILL*-*ELL* was developed with school-aged English as a second language (ESL) learners in the United States, its reliability, validity, and factor structure cannot be assumed to generalize directly to college learners in an EFL context. As a secondary purpose, the study examined whether selected, theory-guided cross-loadings provided psychometric evidence consistent with Oxford's claim that some strategies may serve more than one function. The development of the *SILL*-*SEFL* was not treated as a separate, independent aim, but as the outcome of this cross-cultural validation and model refinement process. Accordingly, the study addressed three related questions: First, whether the six-factor structure of the *SILL*-*ELL* could be retained for Taiwanese college EFL learners; second, what theoretically and statistically justified modifications were needed to obtain an acceptable measurement model; and third, whether the resulting cross-loading pattern was consistent with, but not definitive proof of, strategy role flexibility.

These aims are ordered by priority. Cross-cultural validation of the *SILL*-*ELL* Student Form is the primary aim. The 25-item *SILL*-*SEFL* is a by-product of that validation rather than an independent goal. The test of strategy role flexibility through theory-guided cross-loadings is a secondary and exploratory aim, and its results are read as consistent with the role flexibility theory of Oxford (2017) rather than as confirmation of it.

## 2 Literature review

### 2.1 Challenge of defining language learning strategies

The difficulty of classifying language learning strategies arises from the difficulty of defining the construct. Since Rubin's (1975) early work on the “good language learner,” strategies have generally been understood as techniques, thoughts, or actions that learners use to support language learning. Later studies expanded this view by emphasizing goal orientation, problem solving, transferability across tasks and contexts, and self-regulation (Macaro, 2006; Gu, 2012; Oxford, 2017). Despite this shared orientation, researchers have not reached full agreement on what counts as a second language (L2) learning strategy. O'Malley et al. (1985), for example, identified a great number of strategies and noted the lack of consensus regarding the construct, while Dörnyei (2005) characterized learning strategies as highly ambiguous. This definitional uncertainty has contributed to long-standing disagreement about how strategies should be classified.

Oxford's (2017) synthesis helps move the discussion forward by treating L2 learning strategies as complex, dynamic, goal-directed thoughts and actions that learners select in specific contexts to regulate cognitive, affective, social, and other aspects of learning. This definition is important for the present study because it implies that strategies may not have fixed, single functions. A strategy may serve different purposes across tasks or contexts, and learners may combine strategies in clusters or chains. Thus, the definitional debate leads directly to a psychometric question: Should a strategy item be forced to represent only one factor, or can it legitimately relate to more than one strategy category? The following section addresses this question by considering the distinction between simple and complex structure.

### 2.2 Classification of strategies: the dilemma between simple and complex structures

In psychometrics, a “simple structure occurs when each item is strongly linked to one and only one factor” (Furr, 2018, p. 94). Such a structure makes the factor in question more straightforward to interpret and clarifies the connections among items or variables. In the *SILL* of Oxford (1990)—often considered foundational—each of its 50 items (Version 7.0) is explicitly aligned with one factor. For example, Item 5, “I use rhymes to remember new English words,” is theoretically grouped under Factor 1 (Memory Strategies), whereas Item 35, “I look for people I can talk to in English,” is placed under Factor 4 (Metacognitive Strategies). Over the past four decades, adhering to a simple structure has guided researchers as they constructed strategy theories (e.g., O'Malley and Chamot, 1990; Oxford, 1990; Rubin, 1981), identified underlying strategy factors (Yang, 1999; see Oxford and Burry-Stock, 1995 for an early review), and validated instruments used to measure strategies (e.g., Ardasheva and Tretter, 2013; Hsiao and Oxford, 2002).

As early as the 1980s, scholars such as O'Malley et al. (1985) confronted the decision to employ a simple structure or a complex structure in categorizing strategies. When the direct and indirect classification scheme of Rubin (1981) was used to group cognitive strategies, “mutually exclusive categories” proved unattainable because “some strategies appeared in more than a single grouping” (p. 32). From a psychometric standpoint, “mutually exclusive categories” indicates the preference for a simple structure.

This simple structure also stands in line with Rubin's (1981) division between direct and indirect learning strategies. In fact, O'Malley and Chamot (1990) proposed a tripartite classification of learning strategies consisting of cognitive, metacognitive, and socio-affective strategies, which are all simplified procedures based on the premise of content-knowledge (right) and control (wrong). One of the clearer illustrations of the adoption of simple structure is manifested by Oxford's *SILL* (1990). There are two general dimensions to the *SILL*, which is made up of two large categories: Direct Strategies (memory, cognitive, and compensation components) and Indirect Strategies (metacognitive, affective, and social components).

The simple structure approach has been widely used in research on language learning strategies. Based on the data, factor analysis was conducted to construct the theoretical framework and factor structure of the *SILL* of Oxford (1990), and simple structure practice was continued in later studies that aimed to verify its factor structure. For example, a confirmatory factor analysis conducted by Hsiao and Oxford (2002) found that none of the 15 models being tested fit the data well. Likewise, Ardasheva and Tretter (2013) validated a 28-item *SILL*-*ELL* Student Form, keeping the six-dimensional nature of the *SILL* intact. Both of these studies used a simple structure framework to guide their evaluations and adaptations.

In contrast, studies that were based on exploratory factor analysis or principal components analysis to detect the *SILL*'s factors rarely found the six-factor structure described by Oxford (1990). Most of these analyses led to different solutions. Oxford and Burry-Stock (1995), for instance, based on analyses of data collected in Puerto Rico, Taiwan, the People's Republic of China, Japan, Egypt, and the US, reached nine factors. In pursuit of the most suitable factor structure, Oxford and Burry-Stock followed the “simple structure” principle (p. 14).

Adoption of a simple structure is also visible in a broad range of studies that propose different factor solutions for the *SILL* (Oxford, 1990). These include a one-factor model (Heo et al., 2012), models with three or five factors (Danko and Dečman, 2019), models with six modified factors (Papadopoulou et al., 2018; Saks and Leijen, 2018), and configurations comprising 13 or 15 factors (Robson and Midorikawa, 2001). These results do not support the six-factor model put forth by Oxford (1990). Taken together, these findings call into question the empirical adequacy of the six-factor model proposed by Oxford (1990) and suggest the need for further examination of its theoretical and measurement foundations. To contextualize these mixed findings, Table 1 presents a *selected* summary of prior *SILL* validation and short-form studies, outlining their samples, analytical methods, factor structures, and main results, and identifying gaps addressed in the present study.

**Table 1:** Summary of key *SILL* validation studies.

Study	Sample	Method	Factors	Key findings
Oxford and Burry-Stock (1995)	Multiple countries, various ages	EFA	9	Factor structure varied across samples
Hsiao and Oxford (2002)	517 college EFL (Taiwan)	CFA	6	Tested 15 models; none fit well
Robson and Midorikawa (2001)	188 college EFL (Japan)	EFA	13–15	Failed to replicate six-factor structure
Ardasheva and Tretter (2013)	1,057 K-12 ESL (USA)	CFA	6	28-item *SILL*-*ELL*; simple structure
Papadopoulou et al. (2018)	Adult EFL (Greece)	CFA	6	Modified six-factor; simple structure
Current study	509 college EFL (Taiwan)	CFA	6	25-item *SILL*-*SEFL*; cross-loadings tested

EFA, Exploratory factor analysis; CFA, Confirmatory factor analysis. The current study is distinguished by explicitly testing strategy role flexibility through cross-loadings rather than constraining items to simple structure.

In light of these unresolved issues, if a complex structure is adopted in validating the underlying factors of language learning strategies, the outcomes may provide a fuller perspective on how strategies are used in language learning. This approach may shed additional light on the recently proposed strategy role flexibility theory of Oxford (2017) and its practical value. The study described here has been designed to investigate the strategy role flexibility hypothesis directly.

### 2.3 Strategy role flexibility and measurement modeling

Oxford (2017) has recently put forth a significant argument that

“strategy categories are not rigid but are instead flexible and permeable” (p. 141).

She goes on to emphasize the need for recognizing the intricate nature of learning strategies, which can assume multiple roles. To address the longstanding theoretical ambiguity that has persisted in the literature (e.g., Dörnyei, 2005; Dörnyei and Ryan, 2015; Dörnyei and Skehan, 2003), she proposes strategy role flexibility and raises several questions:

As predicted, [strategy] classification conflicts did ensue … However, what if classification conflicts reflected authentic differences in how strategies operate, including the changing roles a given strategy can have within a single task and across tasks? … it is literally impossible to classify a given strategy … into one and only one particular slot, such as metacognitive, cognitive, or affective? (p. 141)

Oxford's (2017) proposal for flexibility in strategy categories challenges the prevailing notions of language learning strategies. In specific, it presents a more dynamic and adaptable approach to how strategies are used and interpreted. In other words, the proposed view emphasizes the multifaceted roles of strategies within language learning processes as well as their application in specific tasks.

Researchers who address statistical perspectives on language learning strategies often discuss multiple roles of strategies and consider flexible, permeable categories that can connect particular strategies to more than one underlying factor. However, many analyses of the *SILL* (Oxford, 1990) do not incorporate Oxford's (2017) concept of role flexibility. In addition, second language acquisition research outside this domain rarely explores cross-loadings and multiple influences, which restricts comprehensive evaluations of strategy usage and may lead to incomplete theoretical models. Alamer and Marsh (2022) tackled this research gap in their dualistic model of passion and showed that items representing latent constructs frequently overlap. The authors observed that item cross-loadings occur regularly in psychological measurement, suggesting the inevitability of some overlap in constructs. When considered in light of the present study, this insight suggests that certain language learning strategies may fall under the purview of more than one strategy factor.

Researchers in other fields, particularly those working with structural equation modeling, have increasingly recognized the value of including cross-loadings between items and factors beyond their intended targets (e.g., Asparouhov and Muthén, 2009; Asparouhov et al., 2015; Brown, 2015; Byrne, 2006; Hsu et al., 2014; McNeish and Wolf, 2023; Morin et al., 2016). Furthermore, as discussed above, psychological constructs frequently exhibit overlap (Marsh et al., 2009), which raises questions about the appropriateness of rigid factor structures. Given these concerns, we consider this approach seriously, particularly in view of strategy theories such as those proposed by Oxford (2017), which advocate for such flexibility.

### 2.4 Strategy use: instruction, context, and role flexibility

Context and teachability, two essential features in Oxford's (2017) comprehensive definitions of L2 strategies, are important variables predictive of strategy use. Learners' strategy use tends to vary with, among others, contextual factors (e.g., learning ESL in the U.S. vs. learning EFL in Taiwan; Griffiths, 2018). Learning context is often associated with its culture in which the learning takes place. Liu and Rao (2024) found that Australian and East Asian students reported using different strategies, with the former using strategies of relevance to self-realization, and the latter using strategies related to working hard and perseverance. In Politzer and McGroarty (1985) early study in which students were grouped according to their cultural background, Hispanics were found to use all three types of the learning strategies significantly more frequently than their Asian counterparts.

With regard to strategy use, quality could be more important than quantity, and this difference, which is probably associated with strategy role flexibility (Oxford, 2017), has received empirical support. In Plonsky's (2011) meta-analysis in response to the mixed findings regarding the effectiveness of strategy-based instruction, a pooled effect size of *d* = 0.49 was derived from 95 independent samples. Moreover, this medium effect size was observed to vary according to factors such as the frequency and type of strategies used, the learning context (i.e., ESL or EFL), and the duration of the intervention.

Having a closer examination regarding the frequency of strategy use, subgroup analyses of Plonsky (2011) found that studies with more than eight strategies produced a notably smaller effect size (*d* = 0.28, 95% *CI* = [0.15, 0.41]) than studies with fewer than eight strategies (*d* = 0.69, 95% *CI* = [0.64, 0.74]). The counterintuitive difference in the observed results suggests that the frequency of strategy use may not provide insights into their overall quality. Learners in the latter group may employ approaches serving multiple functions, whereas those in the former group could rely on strategies with narrower roles. Four effective compensation strategies, for example, could play 12 distinct roles, whereas six cognitive strategies might address only six functions. Such a possibility remains central to the strategy role flexibility hypothesis of Oxford, in broader considerations of language learning.

The varying effectiveness of strategy instruction becomes evident when we consider different learning contexts. The results of this moderator (Plonsky, 2011) showed a notably significant and large effect size for studies conducted in a second language context (*d* = 0.84, 95% *CI* = [0.71, 0.96]) than for studies conducted in a foreign language context (*d* = 0.46, 95% *CI* = [0.41, 0.51]). The different effects between the two contexts emphasize the significance of considering contextual factors, confirming Oxford's (2017) assertion that

“[a]ny relationship between strategy instruction… and its outcomes can only legitimately be understood in reference to the contexts in which they occur” (p. 309).

In light of the aforementioned theoretical underpinnings, this study is conducted to cross-culturally validate the *SILL-ELL Student Form* of Ardasheva and Tretter (2013). As their scale was based on school-aged ESL learners in a Midwestern district in the United States, whether it is also reliable and valid in an EFL context awaits empirical verification. An additional important purpose of this study is to test Oxford's (2017) strategy role flexibility hypothesis.

### 2.5 Positioning the current study: comparisons with prior *SILL* short forms

To improve practicality, several short forms of Oxford's (1990) 50-item *SILL* have been developed; among them, the 28-item *SILL*-*ELL* (Ardasheva and Tretter, 2013) retains the original six-factor structure. Building on this work, we validate the instrument with college EFL learners and test Oxford's (2017) strategy role flexibility by evaluating a CFA model with targeted, theory-guided cross-loadings.

## 3 Methodology

### 3.1 Participants and research context

To validate the *SILL-ELL Student Form* (Ardasheva and Tretter, 2013) developed in an ESL context, we recruited 529 college EFL learners in Taiwan. After 20 cases with missing data were removed, the final sample consisted of 509 students, with more males (70%) than females (30%). These students were aged between 18 and 20 years, majoring in engineering, fisheries science, maritime science, and management, and had learned English for 6 years as a school subject during their junior and senior school years. When the data were collected, they were taking a required first-year English course conducted in both English and Mandarin Chinese. Most of these learners had achieved the intermediate-level of the *General English Proficiency Test* of Taiwan (for a review of this test, see Roever and Pan, 2008). Participants in this study differ from those in Ardasheva and Tretter (2013) in several ways. By way of comparison, whereas participants in the Ardasheva and Tretter study were elementary (61.6%) or high school (38.4%) students, with a mean age of 12.21 years (SD = 2.80 years), those in the current investigation were college students and were in their late teen (mean age = 19.24 years; SD = 0.83 years; range = 18–20 years). In addition, students in Ardasheva and Tretter's study were learning ESL, that is, as a language that is used in daily communication by members of community who use the language. In this context, learners have more exposure to the language outside the classroom. On the other hand, students in the current investigation were learning EFL, that is, as a language that is typically learned in the classroom context, and the language being learned is not the dominant language spoken and used by members of the community. Students in this context have limited exposure to the language outside the classroom in comparison to ESL learners.

Sampling followed a convenience procedure. We recruited intact classes of first-year students who were taking a required English course, which gave access to a large and demographically consistent pool of intermediate college EFL learners. This procedure suited the validation aim, because instrument validation depends on a sufficiently large and homogeneous calibration sample rather than on a probability sample of a broader population.

### 3.2 Procedures

Participants responded to a Chinese version of the questionnaire during regular class time. The questionnaire included items for collecting data on individual-difference variables (e.g., attitude, motivation, self-concept, and anxiety) and several variables associated with learning strategies, such as knowledge about their use, perceived difficulty about using them, and their effectiveness, but only data for L2 learning strategy use were analyzed in the current validation. Written informed consent was obtained from all participants before they completed the questionnaire. Participants were then given instruction, in Chinese, about how to respond to the questionnaire, and were ensured that (a) the survey study was not a test and was not associated with their instructors or grade, (b) there was no right or wrong answer in the questionnaire, (c) they were not required to give identifiable information (no names, student IDs, or email addresses were collected), (d) the collected data were analyzed at the aggregate level, and (e) the collected data would be maintained with utmost confidentiality. All data files were stored on password-protected computers accessible only to the research team.

Prior to the main administration, content and translation validity were addressed through several steps. First, the item content was drawn from Oxford's (1990) *SILL* and Ardasheva and Tretter's (2013) *SILL*-*ELL* Student Form, both of which were developed to represent Oxford's six strategy categories. Second, the English items were translated into Chinese by the first author, who holds a Ph.D. in Teaching English as Foreign Language and is bilingual in English and Mandarin Chinese. The translated version was then back-translated into English by a colleague with a doctoral degree in TESOL who is also fluent in both languages. The original, translated, and back-translated versions were then compared item-by-item to evaluate semantic equivalence, conceptual consistency with the intended strategy category, cultural appropriateness, and clarity. Discrepancies were resolved through discussion. Third, the Chinese version was cognitively pretested with 10 students from the target population, who provided feedback on item clarity and naturalness. The pretest was not intended to estimate reliability, and no reliability coefficient was calculated because the small number of students would not provide a stable reliability estimate. Reliability and validity evidence were instead evaluated using the full validation sample.

### 3.3 Instrumentation

Students' use of L2 strategies was measured on Oxford's (1990) *SILL* (version 7.0) designed specifically for EFL or ESL learners, and the items for the current study were measured on an eight-point scale anchored by “Never use it” (1) and “Use it very often” (8). This version of the *SILL* includes 50 specific strategies theorized to assess six major types of learning strategies: Memory (Items 1–9), Cognitive (Items 10–23), Compensation (Items 24–29), Metacognitive (Items 30–38), Affective (Items 39–44), and Social (Items 45–50), and the current validation was based on 28 of the 50 strategies that were included in the *SILL-ELL Student Form* of Ardasheva and Tretter (2013). Their validation study reports a six-factor solution consistent with Oxford's strategy theory, and the model implied in their *SILL-ELL* evidences a substantial improvement in the assessment of strategies after removing 22 of the 50 items. The 28 items remaining in the model include seven, five, five, four, three, and four specific strategies for the six respective strategy factors in Oxford. Specifically, the 28 strategies of Ardasheva and Tretter correspond to Oxford's *SILL* Items 6, 5, 7, 2, 3, 4, and 9, in the order given, for memory strategies; Items 16, 18, 19, 21, and 23 for cognitive strategies; Items 25, 26, 27, 28, and 29 for compensation strategies; Items 31, 32, 33, and 38 for metacognitive strategies; Items 41, 43, and 44 for affective strategies; and Items 45, 46, 47, and 48 for social strategies.

### 3.4 Analytical plan

Before using confirmatory factor analysis to validate the framework of Ardasheva and Tretter's (2013) *SILL-ELL Student Form*, we have to hypothesize the number of factors relating to which items should load on which factors and whether to allow the factors to be correlated. The analytical processes involve five basic steps: (a) specification, (b) identification, (c) estimation, (d) testing, and (e) modification.

We used a confirmatory factor analysis framework rather than exploratory structural equation modeling because the study tested a small set of specific, theory-derived secondary loadings rather than searching for structure. Confirmatory factor analysis does not require a strict simple structure. Cross-loadings can be specified when theory predicts them, and this is how strategy role flexibility was operationalized in the present study. Exploratory structural equation modeling, in contrast, frees every cross-loading and raises the risk of capitalizing on chance. A constrained, theory-guided confirmatory model therefore offers a more direct and falsifiable test of the predicted secondary loadings. This confirmatory approach cannot detect unanticipated structure, so the results are read as consistent with the role flexibility theory of Oxford (2017) rather than as proof of it.

#### 3.4.1 Model specification

For the first step, the model to be tested was based on the measurement model from Ardasheva and Tretter's (2013) *SILL-ELL Student Form*. In this model, the six strategy factors, treated as latent variables, were hypothesized to be intercorrelated. These six latent variables were the causes of the 28 strategies treated as manifest variables.

#### 3.4.2 Model identification

In the second step, we had to make sure that the measurement model can be identified, that is, verifying that the number of parameters estimated does not exceed the number of data points. With 28 manifest variables included in the model, the number of data points was 406 (=28^*^(28+1)/2), and we estimated 71 parameters: (a) 28 regression weights (i.e., loadings) between the latent strategy factors and the items, (b) 15 covariances among the six latent factors, and (c) 28 variances for the 28 error terms. We did not estimate the variances for the six latent variables; they were fixed at 1.0 for identification purposes. The measurement model specified here had 335 degrees of freedom (=406–71), and, therefore, can be identified.

#### 3.4.3 Model estimation

Proceeding to the third step, we used the SAS Proc CALIS procedure with maximum likelihood method to estimate the parameters (SAS Institute Inc, 2016), and the estimation processes were based on covariance matrix rather than on the correlation matrix, as (Cudeck 1989) recommended. If the assumption of multivariate normality does not hold, we would make use of robust statistics, for example, by invoking the “Method=MLSB” option in SAS to incorporate Satorra-Bentler scaled corrections (Satorra and Bentler, 1988).

#### 3.4.4 Model testing

In the fourth step, we tested whether the hypothesized measurement model provided an appropriate fit to the data. Model fit was evaluated using multiple indices rather than a single cutoff: The comparative fit index (CFI; Bentler, 1990), the Tucker–Lewis index (TLI), the root mean square error of approximation (RMSEA; Browne and Cudeck, 1992; Steiger, 1990) with its 90% confidence interval, and the standardized root mean residual (SRMR; Hu and Bentler, 1999). Following common SEM practice, CFI and TLI values around 0.90 were interpreted as indicating acceptable fit and values around 0.95 as indicating good fit, whereas RMSEA and SRMR values of 0.08 or below were interpreted as acceptable and values around 0.05 as indicating close fit. These cutoff values were treated as interpretive guidelines rather than rigid decision rules because fit indices can be affected by sample size, number of items, model complexity, and factor correlations. Therefore, final model evaluation was based on the overall pattern of fit indices, theoretical interpretability, absence of improper solutions, and cross-validation in the holdout subsample.

These cutoff values for the CFI, TLI, RMSEA, and SRMR were set a priori on the basis of established guidelines rather than adjusted to the present data.

#### 3.4.5 Model modification

As the final step, we would modify the model to improve the model, guided by theoretical and empirical considerations. Empirically, the modifications partially relied on both the multivariate Lagrange Multiplier (LM) test and the Wald test (Wald, 1943). The LM test evaluates whether a new parameter can be added to the model to produce a smaller χ^2^ (i.e., a better model). A significant *p* value associated with a newly added path would indicate that adding a path can significantly improve the model (Buse, 1982; Bentler and Chou, 1987). On the other hand, the Wald test examines whether removing a parameter (i.e., fixing the path at zero) would not significantly decrease the model performance. Generally, this statistic is used to identify unimportant paths that can be deleted without significantly increasing the χ^2^ value.

In addition to the LM test and Wald test, we also considered the magnitude of factor loadings.

“In applied research, factor loadings greater than or equal to .30 or .40. are often interpreted as *salient*” (Brown, 2015, p. 27).

Thus, we used 0.30 as a criterion for factor loadings; a strategy item that did not cross-load and its loading did not reach the 0.30 level would be deleted. However, small cross-loadings, if significant, will be retained because

“forcing even small cross-loadings to zero was detrimental to empirical identification, estimation bias, power and Type I error rates” (Zhang et al., 2023, p. 115).

All of the modifications were made one at a time as guided by a conceptual and empirical rationale.

Model modifications (re-specifications) were driven by an important theoretical feature of learning strategies that Oxford (1990) has repeatedly cautioned:

“… particular strategies could be viewed as related to more than one strategy category” (Hsiao and Oxford, 2002, p. 379).

Perhaps no strategy researcher has so far tested this role flexibility when uncovering the *SILL*'s factor structure. Outside the field of L2 strategies, Alamer and Marsh (2022) highlight the importance of cross-loadings in their dualistic model of passion. They argue that psychological items of latent constructs cannot perfectly represent their intended factors, and, therefore,

“item cross-loading should be expected and accepted” (p. 1479).

In other disciplines, structural equation modeling researchers have paid increasing attention to the issue of cross-loadings (e.g., Asparouhov and Muthén, 2009; Asparouhov et al., 2015; Brown, 2015; Byrne, 2006; Hsu et al., 2014; Morin et al., 2016). We, therefore, took this approach into serious consideration when empirical evidence suggested such a need, given that psychological constructs tend to overlap (Marsh et al., 2009).

To reduce the risk that modification indices would simply capture idiosyncratic features of the calibration sample, we treated them as diagnostic information rather than as automatic decision rules. A parameter was added or removed only when three conditions were met: First, the change was consistent with Oxford's role flexibility framework; second, the change improved model fit or parsimony in a meaningful way; and third, the revised specification could be evaluated in the holdout subsample. We also avoided adding correlated residuals solely to improve model fit, because such parameters are often difficult to interpret in a strategy-classification model. Thus, the final model should be understood as a theoretically constrained and cross-validated revision rather than as a purely data-driven search. In this series of model modifications, there was the issue of capitalizing on chance characteristics of the sample data (Tabachnick, and Fidel, 2013). Before testing Ardasheva and Tretter's (2013) factor structure in the *SILL-ELL Student Form*, we randomly split the entire sample (*N* = 509) into a calibration subsample (*N*_1_ = 259) and a holdout subsample (*N*_2_ = 250) using a SAS random number generator. Data from the calibration subsample were used to test the initial measurement model of strategy use, and the final, revised model was then cross-validated on the holdout subsample (Cudeck and Browne, 1983; MacCallum et al., 1992).

## 4 Results

### 4.1 Data screening

We first examined whether the data were normally distributed. There was little evidence of any substantial univariate kurtosis or skewness (see **Appendix A**), with most values being between −3.0 and 3.0 (Kline, 2011). For the entire sample, the values for kurtosis ranged between −1.21 and 0.78, and the values for skewness between −0.86 and 1.31, with the exception of Item 23 which exhibited a kurtosis value of 4.48 and a skewness value of 2.20. To overcome the normality problem and multivariate normality problem that may result from the behavior of Item 23, subsequent confirmatory factor analysis was conducted with Satorra and Bentler's (1988; 1994) scaling corrections for computing the Satorra-Bentler chi-square value (S-B χ^2^) and related robust statistics.

### 4.2 Initial validation using the calibration subsample

The measurement model of Ardasheva and Tretter's (2013) *SILL-ELL Student Form* was subjected to confirmatory factor analysis. We used SAS Proc Calis procedure with maximum likelihood solution (SAS Institute Inc, 2016) and invoked Satorra and Bentler's (1988) method to obtain robust statistics for the model. With 259 participants in the calibration subsample for modeling the relationships between the 28 strategy items and six latent variables, the sample size could be a concern. A power analysis was thus conducted using a syntax adopted from MacCallum et al. (1996). We specified *N* = 259, alpha level = 0.05, degrees of freedom = 335 (for the initial theoretical model), and alternative RMSEA = 0.08. A power of 1.0 was obtained based on the results from testing the initial hypothetical model, suggesting that the model had sufficient statistical power to estimate what actually exists in the population.

**Figure 1:**
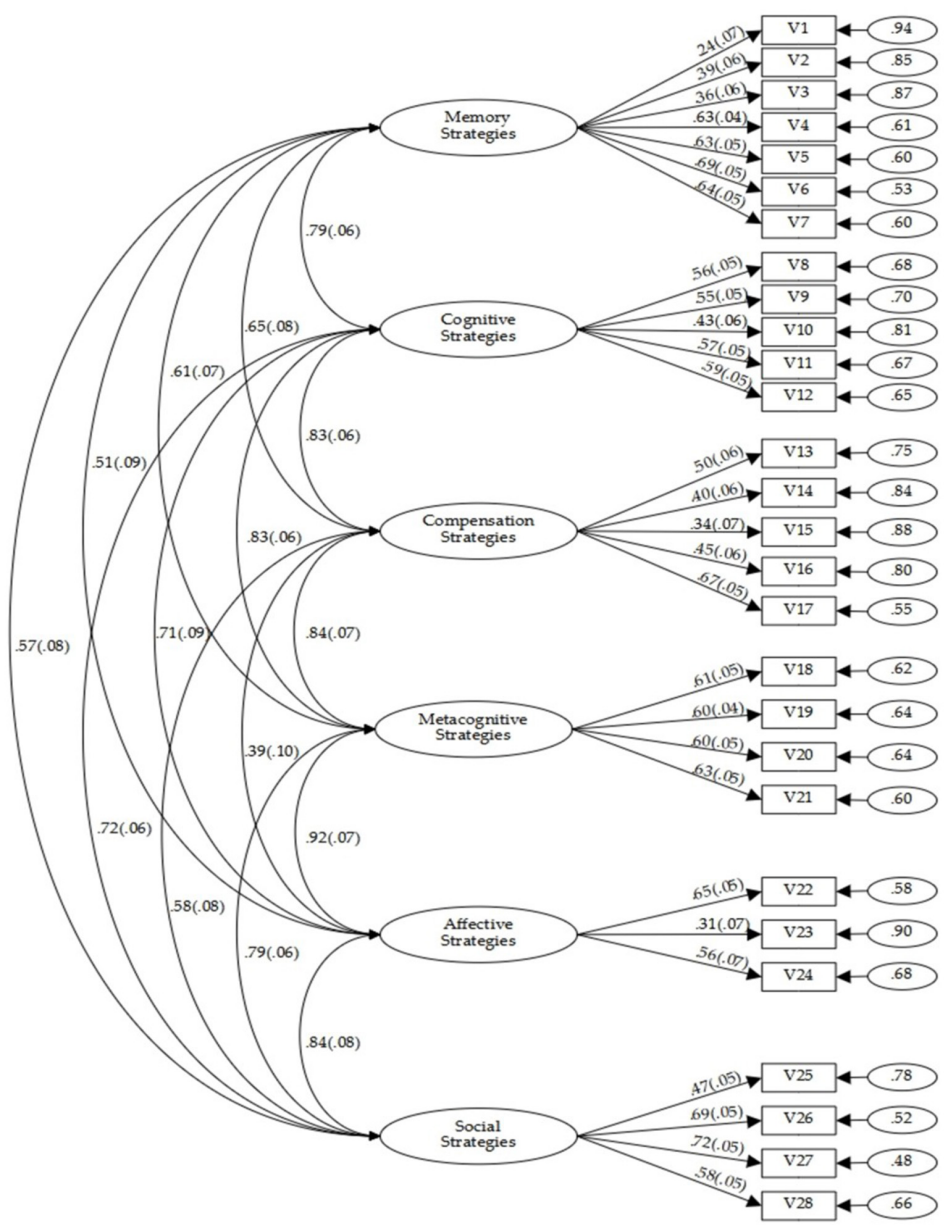
Initial confirmatory factor analysis results for the *SILL-ELL Student Form* tested on the calibration sample: inter-factor correlations, standardized factor loadings, and error variances (*N*_1_ = 259).

Confirmatory factor analysis results for the initial theoretical model are reported in Table 2 and Figure 1.

**Table 2:** Values of Fit Statistics for Models of the *SILL-ELL Student Form*.

Model	χ^2^	S-B χ^2^	*df*	S-B χ^2^/*df*	SRMR	RMSEA (90% CI)	CFI
Null model	2306.06	2079.09	378	5.50	–	–	–
Theoretical model^a^	714.22	612.60	335	1.83	0.071	0.056 [0.049, 0.063]	0.837
Final model-C^a^	433.25	376.42	256	1.47	0.058	0.042 [0.033, 0.051]	0.926
Final model-H^b^	451.97	395.65	256	1.55	0.059	0.046 [0.037, 0.055]	0.902
Final Model-CH^c^	561.67	483.10	256	1.89	0.049	0.042 [0.036, 0.047]	0.925

All chi-squared statistics were significant, p < 0.0001; χ^2^ = Chi-square for conducting χ^2^ difference tests (Anderson and Gerbing, 1988; Steiger et al., 1985); S-B χ^2^ = Satorra-Bentler chi-square (Satorra and Bentler, 1994); df = degrees of freedom; SRMR = standardized root mean residual (Hu and Bentler, 1999); RMSEA, root mean square error of approximation (Steiger, 1990); CI, confidence interval; CFI, comparative fit index (Bentler, 1990).

^a^*N*_1_ = 259 (calibration subsample);

^b^*N*_2_ = 250 (holdout subsample);

^c^N = 509 (entire sample).

As reported in Table 2, the S-B χ^2^ value for the theoretical model (M1) was 612.60, with 335 degrees of freedom, *p* < 0.0001. This value represented the discrepancy between the unrestricted sample covariance matrix and the hypothetical covariance matrix, suggesting an unacceptable model fit to the data. However, because the chi-square test is sensitive to the sample size, we also examined the SRMR and RMSEA (both being absolute indices) and the CFI (a relative fit index) for the model. For the theoretical model M1, although the SRMR value of 0.071 was smaller than 0.08, both the RMSEA value (=0.056) exceeded 0.05 and the CFI value of 0.837 was much smaller than 0.90, an indication that this model was not an appropriate representation of the data. Note that the 90% confidence interval for the RMSEA ranged between 0.049 and 0.063, which represents a good degree of precision.

Additional information about the model is diagrammed in Figure 1. Although all the factor loadings were statistically significant (*p* < 0.01), their values ranged widely from 0.24 (V1) to 0.72 (V27). This was evidence that a relatively small amount of variance was explained for some of the items, for example, only 6% for V1, 12% for V15, and 10% for V23. Taken together, the combined evidence revealed sources of model misfit that should be identified to improve the model.

### 4.3 Model modification using the calibration subsample

We used CFA with targeted, theory-guided cross-loadings. Unlike exploratory structural equation modeling (ESEM), which freely estimates all cross-loadings, Oxford's (2017) framework implies that only specific strategies should exhibit secondary loadings. The resulting cross-loading structure was cross-validated in the holdout sample and was theoretically interpretable.

To identify sources of model misfit for the college EFL subsample, we invoked the SAS “modification” option to provide modification indices for assisting in how the model can be changed with regard to factor loadings (i.e., regression weights) and correlation of error variances. After a series of modifications, V1 (using flashcards to remember new English words) consistently displayed a small loading (< 0.30), which was thus removed from the model. In the second modification, the LM test suggested a largest significant reduction in the chi-square value when a path was added in which V25 (asking the other person to slow down) of Factor 6 was allowed to load on Factor 3. This modification significantly reduced the chi-square by roughly 31.12, as opposed to a chi-square reduction of 27.48 if correlating the error terms for V17 and V25. In the third modification, the test further suggested adding a significant path from Factor 2 to V16 (guessing what the other person will say) which theoretically loads on Factor 3.

Following that, the Wald test was computed to achieve model parsimony. Results from this test would be available only when the statistic identifies a parameter that can be deleted without significantly increasing the chi-square value (i.e., decreasing the model fit). The results suggested the removal of V16 (theoretically a compensation strategy) from Factor 3. Loehlin and Beaujean (2017) warn that one should not “drop every path that is non-significant, especially when the sample size is small” (pp. 68–69).

However, we decided to drop this unimportant path, given that the current sample size was not small and that, after the third modification, the standardized factor loading of V16 on Factor 3 was found to be only −0.06, as opposed to its cross-loading of 0.55 on Factor 2.

After the four modifications, the CFI improved from 0.837 to 0.872 for the initial model, which did not achieve the threshold value of 0.90. Additional effort was thus made to improve model fit and parsimony. Statistical results from the LM test further suggested adding a path from F3 to V21 (thinking about one's progress in learning English) which, in theory, loads on Factor 4. This addition was also theoretically justifiable if we acknowledged the possibility that learners use this metacognitive strategy in a compensatory manner. After this modification, V15 exhibited a behavior similar to the behavior that V1 exhibited earlier, namely, producing a loading smaller than 0.30. As a result, the V15 item was removed for improving model fit and parsimony.

Finally, three additional modifications were made. First, a path was specified from Factor 3 to V9 (reading a passage quickly and then going back and reading it carefully), which resulted in a largest reduction in chi-square and because use of this strategy is clearly compensatory in nature. Second, the results of LM test showed that V14 played an overwhelming role in affecting the model performance. Evidently, this strategy item was found to be associated with five of the 10 largest LM test statistics. Although V14 is theorized as subsumed under Factor 3, the LM test suggested adding a path from any of the remaining five factors, particularly Factors 2 (Cognitive Strategies) and 1 (Memory Strategies), which, if modified, would contribute to the second and third largest reduction in chi-square. In addition to its ambiguous role, V14 was also associated with a relatively low loading in the current analysis. Given these concerns, we removed the V14 item from the model. Third, the LM test suggested adding a path from Factor 3 to V11 (finding the meaning of an English word by dividing it into small parts), which was theoretically sound because learners clearly use this strategy to compensate for their limited vocabulary knowledge.

Overall, with the above model modifications made and justified, the model could be considered acceptable: CFI = 0.926 ≧ 0.90, SRMR = 0.058 ≦ 0.08, and RMSEA = 0.042 ≦ 0.05 (90% CI = 0.033–0.051). The model modifications resulted in removing three strategy items (V1, V14, and V15), respecifying a strategy (V16) from Factor 3 (Compensation Strategies) to Factor 2 (Cognitive Strategies), and adding four cross-loadings for V9, V11, V21, and V25, all of which now load on the Factor 3. All the standardized loadings were statistically significant. For the 21 items that did not cross-load, the loadings ranged between 0.34 (Item 23) and 0.82 (Item 17). Regarding three of the four cross-loaded items, the ranges were between 0.38 and 0.48 for major loadings, and between 0.24 and 0.30 for secondary loadings. One item, V21, deserved special attention because of its large standardized loadings of 1.18 on target Factor 4 and −0.74 on Factor 3. Questions may be raised regarding a standardized loading (i.e., a regression coefficient) of this considerable magnitude. However, as Deegan (1978) demonstrates, “standardized regression coefficients [standardized factor loadings] greater than one can legitimately occur” (p. 873). Jöreskog (1999) also points out “a common misunderstanding” that standardized loadings “must be smaller than one in magnitude and if they are not, something must be wrong” (p. 1).

Because such secondary loadings can also reflect measurement artifacts rather than substantive role flexibility, each cross-loading was retained only when it was consistent with theory, replicated in the holdout subsample, and substantively interpretable.

Substantively, this loading pattern should be interpreted cautiously. Item 21, “thinking about my progress in learning English,” is conceptually a metacognitive strategy, and its large positive loading on the Metacognitive factor is consistent with that interpretation. The negative secondary loading on the Compensation factor should not be interpreted as indicating that progress monitoring is negatively related to compensation strategy use in a simple bivariate sense. Rather, because the Metacognitive and Compensation factors were strongly correlated, the negative coefficient likely reflects a suppression pattern: after the metacognitive component of Item 21 is taken into account, the residual association with compensation is negative. The positive residual variance and successful model convergence indicate that the coefficient does not by itself imply an improper solution; nevertheless, the result suggests that Item 21 is a boundary case and that its secondary compensatory role should be replicated before being generalized.

### 4.4 Model cross-validation on the holdout subsample and analysis on the entire sample

**Figure 2:**
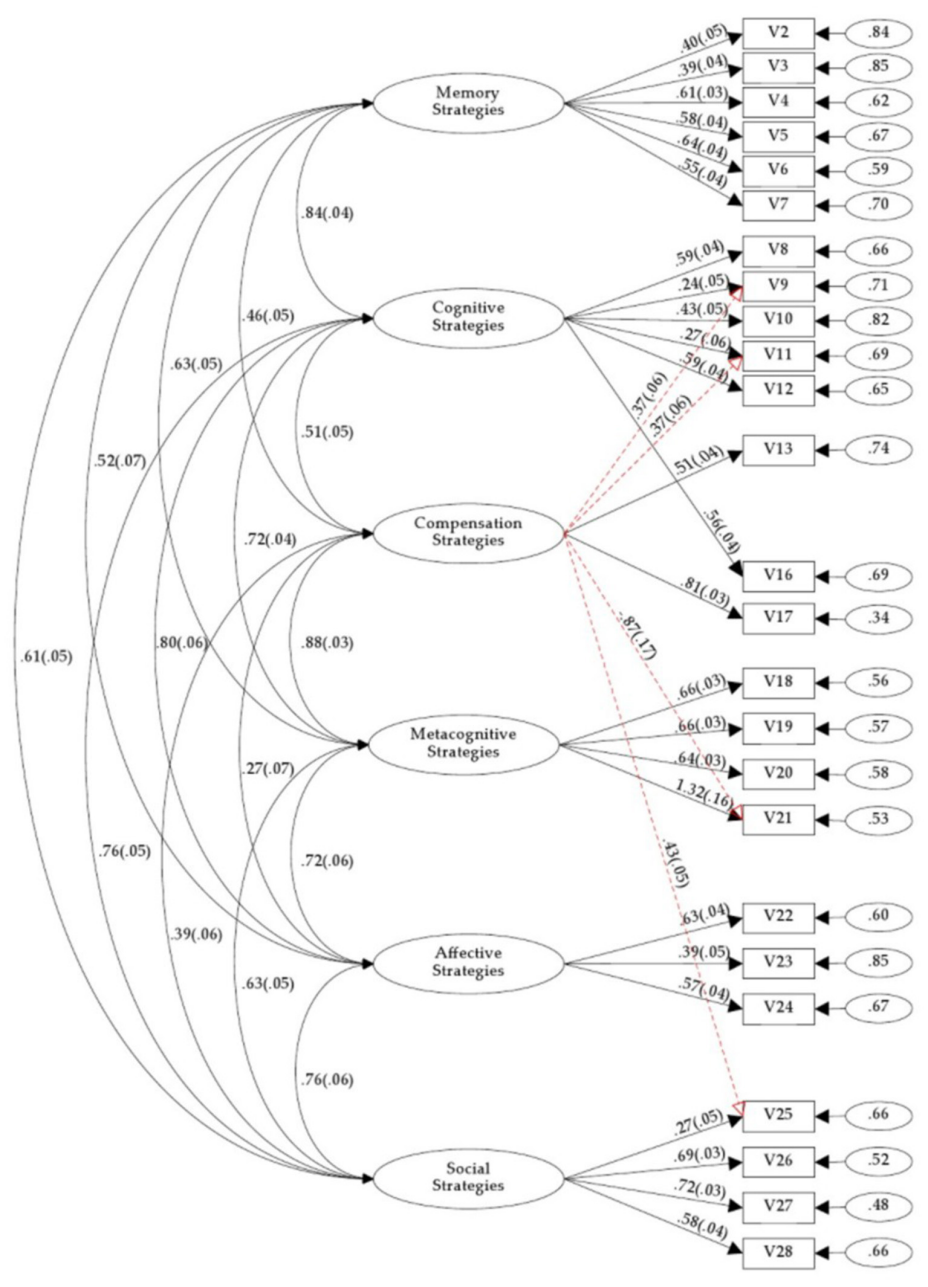
Final confirmatory factor analysis results for the *SILL-SEFL* based on the entire sample: inter-factor correlations, standardized factor loadings, and error variances (*N* = 509).

After the series of model modifications, the risk of capitalization on chance may arise. To address this concern, we further validated the revised measurement model on the holdout subsample. Related fit indices and statistics are reported in Table 2. The CFI index of 0.902 was slightly smaller than the CFI value for the initial, revised model, but its magnitude exceeded 0.90, an indication that the revised model has adequately explained the variance and covariance of the sample data. The SRMR value of 0.059 and RMSEA value of 0.046 both met the criteria of ≦0.08 and ≦0.05, respectively, and were very close to the values (0.058 and 0.042) for the calibration subsample. In addition, all factor loadings were significantly different from zero, and were comparable to those obtained in the calibration subsample. The modified model was successfully cross-validated, and the revised, validated measurement model was fitted to the data of the entire sample (*N* = 509). The estimated fit indices are reported in Table 2 and diagrammed in Figure 2. This analysis produced almost identical results (RMSEA = 0.042 in both analyses; CFI = 0.925 vs. 0.926) and a better (smaller) SRMR index (0.049 vs. 0.058).

Although the holdout results support the stability of the revised model, this procedure cannot fully eliminate the possibility that some parameters reflect characteristics of the present Taiwanese college EFL sample. Cross-validation within the same study guards against the most direct form of capitalization on chance, but it is less stringent than replication in a fully independent external sample from another population. Therefore, the SILL-SEFL should be treated as a promising short-form instrument for comparable college EFL contexts, whereas the item deletions, item reclassification, and compensation-related cross-loadings should be examined in future replications across institutions, proficiency levels, and national EFL settings.

### 4.5 Psychometrical properties of the new scale: *SILL*-*SEFL*

Regarding the psychometric properties based on the entire sample of 509 college EFL learners, results are reported in Table 3 and Figure 2. Because several items exhibited cross-loadings, we used McDonald's (1970) omega reliability (ω), which, unlike Cronbach's coefficient alpha, does not require the assumption of equal factor loadings, or tau-equivalence (McNeish, 2018). The omega reliability coefficient was 0.89 for the revised 25-item *SILL*-*SEFL*. The coefficients were 0.70, 0.69, 0.73, 0.74, 0.55, and 0.71 for Memory, Cognitive, Compensation, Metacognitive, Affective, and Social Strategies factors, respectively. These omega reliabilities are broadly comparable to Cronbach's alpha coefficients reported by Ardasheva and Tretter (2013), namely, 0.90 for the overall scale and 0.77, 0.63, 0.63, 0.72, 0.71, and 0.75 for the six respective strategy factors. Thus, the overall *SILL*-*SEFL* and most of its subscales showed acceptable internal-consistency evidence for research use.

**Table 3:** Omega composite reliability, inter-factor correlations, and χ^2^ for discriminant validity (*N* = 509).

Strategy categories: overall [ω = 0.89]	1 ω = 0.70	2 ω = 0.69	3 ω = 0.73	4 ω = 0.74	5 ω = 0.55^a^	6 ω = 0.71
[0.88, 0.90]^b^	[0.66, 0.74]^b^	[0.65, 0.73]^b^	[0.70, 0.77]^b^	[0.70, 0.77]^b^	[0.49, 0.62]^b^	[0.67, 0.75]^b^
1. Memory	–	582.48	786.79	716.47	627.36	685.52
2. Cognitive	0.84	–	667.92	626.34	576.50	602.93
3. Compensation	0.46	0.51	–	607.69	662.55	758.51
4. Metacognitive	0.63	0.72	0.88	–	591.83	688.18
5. Affective	0.52	0.80	0.27	0.72	–	584.86
6. Social	0.61	0.76	0.39	0.63	0.76	–

Statistics in the upper diagonal were χ^2^ values for conducting chi-square difference tests (Anderson and Gerbing, 1988; Steiger et al., 1985); **ω** **=** omega composite reliability (McDonald, 1970).

^a^The lower reliability for the Affective factor (ω = 0.55) is at least partially attributable to the small number of items (k = 3), as internal consistency estimates are attenuated with fewer items. This subscale should therefore be interpreted cautiously, especially for individual-level score interpretation.

^b^Confidence intervals for omega reliabilities.

The relatively low omega coefficient for the Affective factor (ω = 0.55), however, warrants caution. This estimate is likely attributable in part to the small number of affective items, as the factor included only three items, and the three items still showed meaningful standardized factor loadings of 0.63, 0.39, and 0.57, respectively. Nevertheless, the Affective subscale should not be interpreted as having the same degree of score precision as the other subscales. Scores on this factor are best viewed as brief group-level indicators of affective strategy use rather than precise individual-level diagnostic scores. Therefore, researchers and practitioners should avoid using the Affective subscale alone for high-stakes or individual-level decisions. Future revisions of the *SILL*-*SEFL* should consider adding or refining affective items to improve content coverage and reliability.

With regard to validity, we first assessed the convergent validity of the final scale based on the entire sample. All the factor loadings in Figure 2 were statistically significant for items that did not cross-load, with loadings ranging from 0.39 to 0.81, and also for items that cross-loaded, with major loadings ranging from 0.37 to 1.32 and secondary loadings from 0.24 to 0.87 (in absolute value). These significant loadings indicate that the strategy items can effectively measure their intended strategy factors, and this is evidence that the convergent validity was established.

Next, we tested the discriminant validity. Table 3 reports inter-factor correlations among the strategy factors. Some of the factors were highly correlated (e.g., *r* = 0.88 between Factors 3 and 4), thus raising a concern that the two factors may actually be the same construct and their discriminant validity should be questioned. We then conducted a series of chi-square (χ^2^) difference tests (Anderson and Gerbing, 1988; Steiger et al., 1985) to address this concern, using the χ^2^ statistics in Table 3. With one degree of freedom, the differences in χ^2^ values ranged between 14.83 (=576.50 – 561.67) and 225.12 (=786.79 – 561.67), all statistically significant, *p* < 0.001. These χ^2^ differences provided evidence of discriminant validity for the six strategy factors.

Measurement invariance across gender was evaluated via multi-group CFA. Configural, metric, and scalar invariance were supported based on changes in approximate fit indices (ΔCFI ≤ 0.008; ΔRMSEA = 0.000; Cheung and Rensvold, 2002; Chen, 2007). Given the model's theory-driven cross-loadings and high factor correlations, invariance decisions relied on ΔCFI/ΔRMSEA rather than χ^2^.

To assess the predictive validity of the short 25-item scale (*SILL*-*SEFL*), we examined correlations between learners' strategy scores and their college entrance examination English test scores (Table 4). Overall, the pattern of relationships was comparable to that obtained with the original 50-item *SILL* (Oxford, 1990). In particular, moderate associations were observed for the Cognitive (*r* = 0.33) and Metacognitive (*r* = 0.29) strategy factors, and a smaller but still meaningful association was found for the Compensation factor (*r* = 0.20). Smaller correlations were observed for Memory (*r* = 0.17) and Affective (*r* = 0.15) strategies, whereas the Social factor showed a modest relationship (*r* = 0.19).

**Table 4:** *SILL* and *SILL-SEFL*: correlations between strategy scores and college entrance examination English test scores (*N* = 434).

	*SILL* (Oxford, 1990) and *SILL-SEFL* Categories						
	Memory	Cognitive	Compensation	Metacognitive	Affective	Social	ALL
English test scores	0.17^a^	0.33^a^	0.20^a^	0.29^a^	0.15^a^	0.19^a^	0.31^a^
English test scores	0.19^b^	0.34^b^	0.26^b^	0.27^b^	0.18^b^	0.24^b^	0.31^b^

All correlation coefficients are statistically significant, *p* < 0.01. Subscale scores for the SILL-SEFL were computed by averaging items assigned to each primary factor. Items that exhibited cross-loadings (V9, V11, V21, V25) contributed only to their primary subscale scores.

^a^Based on the SILL-SEFL categories.

^b^Based on the SILL categories.

Notably, these correlations were obtained using a conservative scoring procedure in which cross-loaded items contributed only to their primary subscale scores. Accordingly, *SILL*-*SEFL* subscale scores should be interpreted as indicators of learners' dominant strategy functions rather than as fully independent or mutually exclusive strategy categories. Despite this conservative approach, the *SILL*-*SEFL* demonstrated predictive relationships with English proficiency that closely parallel those observed for the full *SILL*, supporting the criterion-related validity of the short form.

Finally, Table 5 presents a comparison between the current validation and Ardasheva and Tretter (2013) with regard to their standardized factor loadings.

**Table 5:** The present study and Ardasheva and Tretter (2013): comparison of factor loadings.

Items	Factors and strategies	Authors	A&T
	Factor 1: Memory strategies		
1.	Using flashcards to remember new English words.	NA^a^	0.50
2.	Using rhymes to remember new English words.	0.40	0.58
3.	Physically acting out new English words.	0.39	0.52
4.	Using new English words in a sentence to learn them.	0.61	0.61
5.	Connecting the sound and image of a new English word to remember the word.	0.58	0.59
6.	Making a mental picture of a situation where the word might be used.	0.64	0.60
7.	Remember new English words by remembering where their location on the page, on the board, or on a street sign.	0.55	0.57
Items	Factor 2: Cognitive strategies		
8.	Reading for pleasure in English.	0.59	0.45
9.	Skimming an English passage and then going back and reading carefully.	0.24^b^/0.37^c^	0.48
10.	Looking for words in my own language that are like new words in English.	0.43	0.49
11.	Finding the meaning of an English word by dividing it into small parts.	0.27^b^/0.37^c^	0.53
12.	Making summaries of information that I hear or read in English.	0.59	0.60
Items	Factor 3: Compensation strategies		
13.	Using gestures when I can't think of a word during a conversation in English.	0.51	0.54
14.	Making up new words if I do not know the right one in English.	NA^a^	0.57
15.	Reading in English without looking up every new word.	NA^a^	0.32
16.	Trying to guess what the other person will say next in English.	0.56^b^	0.52
17.	Using a word or phrase that means the same thing if I can't think of an English word.	0.81	0.50
Items	Factor 4: Metacognitive strategies		
18.	Noticing my English mistakes and using the mistakes to help me do better.	0.66	0.67
19.	Paying attention when someone is speaking English.	0.66	0.54
20.	Trying to find out how to be a better learner of English.	0.64	0.69
21.	Thinking about my progress in learning English.	1.32^d^/−0.87^c^	0.62
Items	Factor 5: Affective strategies		
22.	Giving myself a reward or treat when I do well in English.	0.63	0.63
23.	Writing down my feelings in a language learning diary.	0.39	0.69
24.	Talking to someone else about how I feel when I am learning English.	0.57	0.70
Items	Factor 6: Social strategies		
25.	Asking the other person to slow down or say it again, if I do not understand something in English.	0.27^e^/0.43^c^	0.65
26.	Asking English speakers to correct me when I talk.	0.69	0.68
27.	Practicing English with other students.	0.72	0.61
28.	Asking for help from English speakers.	0.58	0.69
Overall	Mean of loadings	0.59^f^	0.58

A&T, Ardasheva and Tretter (2013). The overall means were 0.59 for the present study and 0.58 for Ardasheva and Tretter (2013).

^a^Removed during the model modification process;

^b^Loading on Factor 2;

^c^Cross-loading on Factor 3;

^d^Loading on Factor 4;

^e^Loading on Factor 6;

^f^Excluding secondary loadings.

## 5 Discussion

This confirmatory factor analysis cross-culturally validated the 28-item *SILL-ELL Student Form* (Ardasheva and Tretter, 2013) developed based on Oxford's (1990) 50-item *SILL*. While Ardasheva and Tretter's scale provides a reliable and valid tool for ESL learners, teachers, and researcher, the current validation is equally important given that an increasing number of learners worldwide are learning EFL rather than ESL due to the contextual constraints. Validation of Ardasheva and Tretter's scale has led to a short 25-item strategy inventory for college EFL learners, entitled *SILL-SEFL*.

Oxford's (1990) six-factor strategy theory embodied in the *SILL* was previously supported in Ardasheva and Tretter's validation, and is further supported in the current analysis. The short *SILL-SEFL* is found to be both reliable and valid for measuring college EFL learners' strategy use. In particular, although the *SILL-SEFL* has only 25 items, its correlations with EFL learners' college entrance examination English test scores were comparable to the correlations between the 50-item *SILL* scores and the English test scores. Overall, the pattern of associations between strategy use and English test performance was highly similar for the *SILL*-*SEFL* and the original *SILL*. In particular, cognitive and metacognitive strategies showed the strongest relationships with test performance, whereas compensation strategies exhibited a smaller but still meaningful association with proficiency. It should also be noted that the observed association between compensation strategies and English proficiency was obtained using a conservative scoring procedure that assigns cross-loaded strategies only to their primary factors; acknowledging the multifunctional roles of these strategies may yield stronger relationships with learning outcomes.

While the current validation has replicated Ardasheva and Tretter's (2013) six-factor structure of the *SILL-ELL Student Form* theorized in Oxford's *SILL* (1990), there exist notable differences pertinent to the effects that learner or individual-differences variables (e.g., learning contexts, proficiency level, and ethnicity) have on strategy use, and to the strategy classification of Oxford (1990) and her proposal of strategy role flexibility (2017). First, in analyzing the 28 strategies of the *SILL-ELL Student Form*, three of them were removed during the model modification process. The three strategies (i.e., Items 1, 14, and 15 of the *SILL-ELL Student Form*, or Items 6, 26, and 27 of the *SILL*) deserve special attention. Item 1, *I use flashcards to remember new English words*, would less likely be adopted by EFL learners who have achieved beyond the beginning-level proficiency. Intermediate-level students, as those recruited for the current analysis, may judge that there is no such need to this strategy, or the strategy is not effective. Our findings are consistent with the results of Grainger (1997) in that the flashcard learning strategy was among the least preferred strategies by students of Asian background. Thus, the item 1 was removed due to concerns regarding its potential effects for students of Asian ethnicity. In terms of the removal of Item 14, *I make up new words if I do not know the right ones in English*, perhaps this strategy poses a difficulty for EFL learners, which is supported by the results of Hsiao (2004) and MacIntyre and Noels (1996). Item 15, *I read English without looking up every new word*, also was dropped from the model. One explanation is the interaction between learning context and learners' language proficiency. To interpret, the intermediate-level EFL students of the present study would likely find it unnecessary to look up every new word while reading English, and, therefore, the decision to look up unknown words does not seem to play an essential role for these EFL learners. In contrast, the beginning-level ESL learners in the Ardasheva and Tretter (2013) study may perceive this strategy as somewhat necessary, as evidenced by the low loading (λ = 0.32) in their study, even though an ESL context sometimes offers input to help obviate this need. It is possible that the proficiency level of college EFL learners for the current analysis is more powerful than the ESL learning context of the Ardasheva and Tretter study. Consequently, although Ardasheva and Tretter include this strategy in the Compensatory Strategies factor, it is not as salient as other strategies also subsumed in this factor.

The second difference centers on the magnitude of the factor loadings. Although both Ardasheva and Tretter's (2013) study and the current investigation support a six-factor structure of L2 strategies, results in Table 5 showed comparable overall means of factor loadings between the study of Ardasheva and Tretter study (mean loading = 0.58) and the current validation (mean loading = 0.59), if secondary loadings were not considered. However, the loadings of several items vary across the two studies. Items 2 (using rhymes to learn new words; λ = 0.40 vs. λ = 0.58), 3 (acting out new words; λ = 0.39 vs. λ = 0.52), 23 (writing down own feelings in a diary; λ = 0.39 vs. λ = 0.69), 24 (talking to someone else about how I feel when learning English; λ = 0.57 vs. λ = 0.70), and 28 (asking for help from English speakers; λ = 0.58 vs. λ = 0.69) possessed higher loadings in the Ardasheva and Tretter (2013) study than that of our study. On the other hand, higher loadings were found for the latter than for the former with regard to Items 8 (reading for pleasure; λ = 0.59 vs. λ = 0.45), 17 (using a word or phrase that means the same thing; λ = 0.81 vs. λ = 0.50), 19 (paying attention when someone is speaking English; λ = 0.66 vs. λ = 0.54), and 27 (practicing English with other students; λ = 0.72 vs. λ = 0.61). These observed differences in factor loadings present possible evidence that use of specific strategies is contingent on the inherent differences between the two studies, particularly for age (Items 3 and 8), proficiency level (Item 17), learning context (Items 19, 27, and 28), and ethnicity (Item 24). The effects of these variables may also be found in the behavior of Item 16 (guessing what the other person will say next in English). Note that this strategy clearly serves as a compensatory function for Ardasheva and Tretter's (2013) ESL learners (λ = 0.52), but the current analysis suggests that it is more psychologically sound to re-classify this strategy as subsumed under the Cognitive Strategies factor. Are L2 learners using this “guessing” strategy for a compensatory or cognitive purpose? The obvious answer seems to be contingent on the learning context, that is, cognitive for EFL learners and compensatory for ESL learners.

These differences raise an applied question: which short form to use in which context. The *SILL*-*ELL* is best suited to ESL learners similar to its validation sample, whereas the *SILL*-*SEFL* is recommended for intermediate-level college EFL learners with limited out-of-class exposure. Use with other proficiency levels or cultural contexts should be preceded by local validation.

The third difference consists in the item cross-loadings on the latent strategy factors, a phenomenon in L2 research that is inevitable but often is left unexplored when conducting a factor analysis. As stated earlier, scholarly discussions in L2 learning and teaching (e.g., Alamer and Marsh, 2022) and in other areas of inquiry, for example, structural equation modeling (e.g., Asparouhov and Muthén, 2009; Asparouhov et al., 2015), are paying increasing attention to the importance of cross-loadings when studying psychological constructs, because item cross-loadings are permissible (Marsh et al., 2020) and should be accepted (Alamer and Marsh, 2022). As illustrated in Figure 2, model modifications for the current study suggest four cross-loadings for Items 9, 11, 21, and 25, all of which cross-loaded on the Compensation Strategies factor. Item 9, *skimming an English passage and then going back and reading carefully*, originally classified as a cognitive strategy, now also functions as a compensation strategy. Close examination of Figure 2 reveals that this strategy loaded higher on Factor 3 (λ = 0.37) than on the Cognitive Strategies factor (Factor 2; λ = 0.24). This observation is also true of another strategy, Item 11, *finding the meaning of an English word by dividing it into small parts* (λ = 0.27 on Factor 2 vs. λ = 0.37 on Factor 3). In addition, the Compensatory Strategies factor also plays a more important role than the Social Strategies factor for Item 25, *asking the other person to slow down or say it again* (λ = 0.43 on Factor 3 vs. λ = 0.27 on Factor 6). It appears that each of the three strategies plays two roles, that is, compensation and either cognitive or social. This finding strongly supports Oxford's (2017) contention that “strategy categories are not rigid but are instead flexible and permeable” (p. 141). Further, it also justifies Oxford's recent warning “against inflexibility in classifying strategies and in (mis)interpreting strategy classification systems” (p. 142).

The flexibility of classifying L2 strategies may also help ease the difficulty in interpreting strategy use. Several scholars have provided specific examples about this difficulty. For example, Cohen (2018) is uncertain whether “‘interrupting a conversation in the TL' when you are feeling left out” refers to a function that is metacognitive (“monitoring you behavior”), cognitive (“searching for the language material”), social (“checking out whether … appropriate … interrupting the speaker”), or affective (“feeling left out and want to do something”; p. 33). Gao and Hu's (2021) example concerns whether reviewing a list of vocabulary words is a cognitive strategy, or it can also be an affective behavior because learners use this cognitive behavior to manage their anxiety about an upcoming test. Gao and Hu argue that “the boundaries between different strategy categories in *SILL* are often quite blurred” (p. 31). While this point is constructive, we would argue that perhaps it is the flexibility of L2 strategies (Oxford, 2017) that has blurred the functional boundary between strategy factors. Griffiths (2018) raises an additional concern:

If we … accept that metacognitive strategies are those used to ‘supervise or manage … language learning…,' it might be suggested that social and affective strategies are themselves used metacognitively to manage social interaction and affective states, which, in turn, contribute to the management of cognitive engagement. This leaves a more general but well-recognized group of metacognitive strategies…. This line of argument would reduce the basic strategy categories to two…. [c]ognitive strategies … [and] … [m]etacognitive strategies. (pp. 61–62)

Our findings do not appear to support Griffiths's (2018) two-category proposal, nor do they support Dörnyei and Ryan's (2015) proposal of four categories: Cognitive, metacognitive, social, and affective. Attention is drawn to the fact that both proposals exclude the compensation category which, according to Dörnyei and Ryan, refers to communication strategies. The exclusion of the compensation factor is not justified based on our findings. That is, results in Figure 2 show that the Compensation Strategies factor received higher loadings from three of the four cross-loaded items than the Cognitive Strategies (Item 9, λ = 0.37 vs. λ = 0.24; Item 11, λ = 0.37 vs. λ = 0.27) and Social Strategies (Item 25, λ = 0.43 vs. λ = 0.27) factors.

Having a close examination regarding the four cross-loaded items, Item 21, *thinking about my progress in learning English*, deserves further attention. As reported in Figure 2 and Table 5, Item 21 cross-loaded on both the Metacognitive (λ = 1.32) and Compensation (λ = −0.87) Strategies factors. The findings suggest that this strategy serves two functions for college EFL learners, metacognitive and compensatory, apparently with the former exerting more influences than the latter. Notably, the two loadings were in different signs, meaning that the metacognitive and compensatory roles of this strategy seem to work in opposite directions. Moreover, this is a sign that a strategy can compete, in addition to its ability to collaborate, with other strategies. The present investigation presents perhaps one of the first empirical findings pointing out that strategies or roles of strategies can not only collaborate with each other to enhance L2 learning, but they can also compete with each other. As Oxford and Amerstorfer (2018) claimed that L2 strategies “are flexible and fluid” (p. xxiv) with regard to their functions, the current study suggests that when a strategy serves different functions, these functions may represent not only flexibility but in/compatibility of strategy use. Our results, particularly those based on the behavior of Item 21, may explain the “fluctuation in strategy functions when strategies were used either alone, in sequence, or in pairs or clusters” (Cohen and Wang, 2018, p. 169). Therefore, when discussing L2 learners' strategy use, it is not enough to focus only on quantity and quality of strategies; compatibility matters, too.

This interpretation does not imply that Item 21 should be treated primarily as a compensation item in applied scoring. Rather, the item remains substantively metacognitive, while its negative secondary loading suggests that learners' reflection on progress may interact with compensation-oriented strategy use in a more complex way than a simple-structure model would reveal. Thus, Item 21 provides suggestive evidence for role flexibility and possible role incompatibility, but it should also be viewed as one of the most sample-sensitive features of the final model. The importance of strategy compatibility deserves further elaboration. Although the findings surrounding the four cross-loadings highlight the complex “roles or functions a given strategy plays” (Oxford, 2017, p. 141), the role conflict within the same strategy or between different strategies has been rarely addressed in the L2 strategy literature. A common understanding is that good language learners do not necessarily use more strategies than those with lower proficiency. It is possible that lower proficient learners may use strategies that do not suit their personality or learning styles, and the strategies used are incompatible with each other. This incompatibility may help address a concern that Dörnyei (2005) raised:

Thus, the scales in the *SILL* are not cumulative and computing mean scale scores is psychometrically not justifiable. A high score on the *SILL* is achieved by a learner using as many different strategies as possible and therefore it is largely the quantity that matters. This is in contradiction with strategy theory, which has indicated clearly that in strategy use it is not necessarily the quantity but the *quality* of the employed strategies that is important.” (p. 182)

It is reasonable to argue that the compatibility or incompatibility of the adopted strategies have a role to play in the situation that Dörnyei (2005) described. We would encourage future research efforts, particularly qualitative research, to investigate how quantity, quality, and (in)compatibility interact to affect strategy use.

## 6 Conclusion, implications, and limitations

This study has successfully validated the *SILL-ELL Student Form* of Ardasheva and Tretter (2013). The cross-cultural validation has led to a short 25-item scale, *SILL-SEFL*, for college EFL learners. The present results have further justified and highlighted the importance of analyzing

“strategy data to emphasize functions rather than categories and to show rapid fluidity and flexibility of strategies in use” (Oxford and Amerstorfer, 2018, p. 1).

Several pedagogical and methodological implications can be drawn based on the current findings.

Regarding strategy training and instruction, it would be more appropriate to focus on a selected set of strategies that more likely play multiple roles. A good starting point is to place emphasis on strategies that exhibit the compensation function, a function which tends to be shared by most strategies. For classroom practice, prioritizing compensation strategies should be understood as a sequencing and scaffolding principle rather than as an exclusive focus on compensation. Teachers can introduce compensation strategies early because these strategies help learners maintain comprehension and communication when lexical, grammatical, or listening resources are incomplete. For example, reading instruction can include skimming a passage before rereading carefully, using word parts and context to infer meaning, and deciding when exact word-by-word comprehension is unnecessary. Listening and speaking instruction can include asking interlocutors to slow down or repeat, using gestures or paraphrases when a word is unavailable, and using a familiar word or phrase with a similar meaning. These strategies can then be paired with metacognitive reflection, such as asking learners to record which strategy they used, why they used it, whether it helped, and how they might adapt it in a future task.

For curriculum designers, the findings suggest a spiral approach: introduce high-utility compensation strategies in early units, recycle them across reading, listening, and speaking tasks, and gradually connect them with cognitive, social, and metacognitive strategies. Assessment can include short strategy logs, post-task reflections, peer-interaction checklists, or learner self-evaluations that focus not only on how often strategies are used but also on whether learners select strategies that fit the task. Such guidance translates the statistical finding of compensation-related cross-loadings into teachable classroom routines while preserving the need for future intervention research.

At the same time, this pedagogical implication should be interpreted cautiously. The present findings do not provide direct intervention evidence regarding instructional sequencing. Rather, they suggest the hypothesis that strategy instruction might benefit from beginning with compensation strategies, given their apparent multifunctionality and centrality across strategy categories. This hypothesis requires empirical testing through design-based intervention studies that systematically vary the sequencing of strategy instruction and examine learning outcomes. Future research should investigate whether introducing versatile, multi-role strategies first leads to better transfer and more flexible strategy use than traditional sequencing approaches. This proposal is consistent with calls in the strategy instruction literature for empirically grounded pedagogical recommendations (e.g., Plonsky, 2011).

With respect to research methodology, this validation study has demonstrated the usefulness of including cross-loadings in our factor analysis of college EFL learners' strategy use. This practice facilitates a more nuanced understanding of the multiple roles/functions inherent in many L2 strategies. Future research should take this approach into consideration when investigating L2 strategy use and other individual-differences variables, such as L2 motivation and anxiety. With this in mind, L2 strategy research is unlikely to become obsolete and will develop in theoretically and methodologically productive directions.

The generalizability of the findings is restricted in several respects. Participants were intermediate-level college EFL learners from one Taiwanese university, and the sample was male-skewed and concentrated in fields such as engineering, fisheries science, maritime science, and management. Therefore, the results may not generalize to younger learners, older adult learners, beginning or advanced learners, students in other academic disciplines, or learners in ESL contexts where exposure to English outside the classroom is greater. The findings may also vary across EFL settings with different instructional traditions, first-language backgrounds, cultural expectations, and opportunities for English use. This limitation is especially relevant to the cross-loading results because strategy role flexibility is theoretically expected to depend on context. Future studies should replicate the *SILL*-*SEFL* with independent samples from other institutions, countries, proficiency levels, and educational settings to determine whether the item deletions, the reclassification of Item 16, and the compensation-related cross-loadings remain stable.

A further limitation concerns the interpretation of the cross-loadings. Secondary loadings can reflect genuine strategy role flexibility, but they can also arise from measurement artifacts such as overlapping item wording, method effects, or local item dependence. We reduced this risk in three ways. We specified only theory-derived secondary loadings rather than data-driven ones, we required each cross-loading to remain interpretable in the holdout subsample, and we avoided correlated residuals added only to improve fit. Even so, a confirmatory model cannot detect unanticipated structure, and the present design cannot fully separate substantive flexibility from artifact. The cross-loading results, and the boundary behavior of Item 21 in particular, should therefore be treated as suggestive rather than conclusive, and should be re-examined with independent samples and with complementary methods such as exploratory structural equation modeling, bifactor models, and qualitative strategy-use data.

In addition, the study relied on self-report strategy data and a translated version of the instrument. Although translation and back-translation procedures were used, future research should examine whether the same factor structure holds across language versions. Finally, the criterion-related evidence was based on one English examination score; future studies should include multiple proficiency indicators, task-based performance measures, and longitudinal or intervention outcomes.

## Data Availability

The original contributions presented in the study are included in the article/Supplementary material, further inquiries can be directed to the corresponding author.
