# Supplementary material for: Validating a short-form strategy inventory for English as a foreign language learners: testing Oxford's strategy role flexibility theory
Source: Front Psychol. 2026 Jul 22;17:1863208. doi: 10.3389/fpsyg.2026.1863208 (PMC13437348; doi:10.3389/fpsyg.2026.1863208)
Supplement: Supplementary file 1 [file Data_Sheet_1.docx]

Appendix A

Summary statistics of 28 items (upper diagonal for the entire sample, N = 509; lower diagonal for the first subsample, n_1_ = 259)

|  | V1 | V2 | V3 | V4 | V5 | V6 | V7 | V8 | V9 | V10 | V11 | V12 | V13 | V14 |
| --- | --- | --- | --- | --- | --- | --- | --- | --- | --- | --- | --- | --- | --- | --- |
| Mean | 4.21 | 3.17 | 2.45 | 3.81 | 4.99 | 4.53 | 4.39 | 3.24 | 4.95 | 4.30 | 5.23 | 2.95 | 5.28 | 3.47 |
| SD | 2.25 | 2.06 | 1.91 | 1.93 | 2.08 | 2.08 | 2.26 | 1.90 | 2.11 | 2.23 | 2.03 | 1.87 | 2.23 | 2.33 |
| Kurtosis | -1.15 | -0.79 | 0.78 | -0.7 | -0.94 | -1.01 | -1.18 | -0.54 | -1.06 | -1.18 | -0.65 | -0.1 | -0.99 | -1.01 |
| Skewness | 0.11 | 0.6 | 1.31 | 0.4 | -0.34 | 0 | 0.01 | 0.61 | -0.15 | 0.07 | -0.51 | 0.86 | -0.43 | 0.54 |
| V1 | 1 | 0 | .16 | .15 | .16 | .10 | .17 | .14 | .15 | .06 | .05 | .07 | .11 | .08 |
| V2 | -.01 | 1 | .17 | .22 | .28 | .26 | .18 | .25 | .14 | .14 | .08 | .26 | .16 | .19 |
| V3 | .12 | .17 | 1 | .24 | .21 | .21 | .20 | .25 | .07 | .19 | .16 | .23 | .21 | .18 |
| V4 | .17 | .21 | .22 | 1 | .33 | .43 | .32 | .37 | .24 | .13 | .28 | .30 | .17 | .13 |
| V5 | .12 | .35 | .21 | .36 | 1 | .39 | .31 | .23 | .19 | .26 | .26 | .29 | .25 | .17 |
| V6 | .18 | .25 | .18 | .46 | .43 | 1 | .36 | .27 | .19 | .24 | .28 | .28 | .22 | .21 |
| V7 | .20 | .25 | .24 | .39 | .43 | .44 | 1 | .26 | .16 | .22 | .27 | .30 | .21 | .17 |
| V8 | .14 | .19 | .29 | .36 | .23 | .30 | .24 | 1 | .29 | .19 | .19 | .35 | .20 | .23 |
| V9 | .15 | .02 | .06 | .28 | .22 | .25 | .19 | .30 | 1 | .26 | .34 | .28 | .24 | .17 |
| V10 | .05 | .24 | .24 | .16 | .26 | .27 | .18 | .20 | .27 | 1 | .25 | .23 | .15 | .16 |
| V11 | -.02 | .14 | .12 | .29 | .33 | .33 | .31 | .25 | .38 | .28 | 1 | .26 | .29 | .19 |
| V12 | .07 | .22 | .29 | .34 | .33 | .29 | .33 | .37 | .28 | .27 | .34 | 1 | .14 | .24 |
| V13 | .09 | .07 | .22 | .14 | .25 | .25 | .26 | .15 | .23 | .10 | .25 | .13 | 1 | .29 |
| V14 | .09 | .16 | .16 | .15 | .18 | .22 | .21 | .20 | .11 | .15 | .24 | .26 | .23 | 1 |
| V15 | .04 | .10 | .08 | .25 | .11 | .23 | .23 | .18 | .20 | .04 | .20 | .24 | .09 | .12 |
| V16 | .08 | .16 | .25 | .22 | .24 | .27 | .23 | .31 | .21 | .29 | .33 | .30 | .16 | .19 |
| V17 | .04 | .02 | .04 | .23 | .25 | .27 | .15 | .19 | .48 | .12 | .38 | .18 | .40 | .25 |
| V18 | .01 | .15 | .08 | .20 | .29 | .32 | .22 | .26 | .40 | .21 | .43 | .34 | .21 | .19 |
| V19 | .10 | 0 | .03 | .18 | .20 | .22 | .21 | .31 | .32 | .20 | .26 | .27 | .31 | .14 |
| V20 | .23 | .08 | .09 | .27 | .21 | .23 | .17 | .28 | .32 | .23 | .18 | .25 | .27 | .17 |
| V21 | .17 | .20 | .24 | .25 | .25 | .32 | .32 | .29 | .20 | .20 | .27 | .27 | .20 | .31 |
| V22 | .17 | .22 | .10 | .22 | .17 | .22 | .13 | .34 | .20 | .30 | .17 | .24 | .03 | .16 |
| V23 | .16 | .22 | .33 | .15 | .06 | .10 | .17 | .29 | .01 | .15 | .06 | .29 | -.02 | .14 |
| V24 | .19 | .12 | .20 | .11 | .13 | .19 | .2 | .26 | .19 | .25 | .12 | .23 | .12 | .10 |
| V25 | .03 | .04 | .03 | .20 | .13 | .26 | .19 | .24 | .32 | .09 | .26 | .18 | .39 | .22 |
| V26 | .12 | .12 | .26 | .21 | .23 | .18 | .20 | .36 | .23 | .21 | .19 | .32 | .17 | .12 |
| V27 | .20 | .27 | .28 | .30 | .26 | .27 | .30 | .40 | .22 | .17 | .24 | .45 | .15 | .22 |
| V28 | .14 | .13 | .11 | .21 | .14 | .27 | .13 | .24 | .11 | .18 | .15 | .23 | .20 | .15 |
| Mean | 4.11 | 3.16 | 2.50 | 3.73 | 4.81 | 4.56 | 4.36 | 3.09 | 4.97 | 4.25 | 5.12 | 2.84 | 5.30 | 3.50 |
| SD | 2.15 | 2.07 | 1.92 | 1.92 | 2.06 | 2.12 | 2.23 | 1.85 | 2.08 | 2.26 | 2.02 | 1.77 | 2.19 | 2.29 |
| Kurtosis | -1.03 | -0.65 | 0.41 | -0.58 | -0.96 | -1.09 | -1.15 | -0.49 | -1.00 | -1.22 | -0.7 | 0.25 | -0.98 | -0.98 |
| Skewness | 0.25 | 0.68 | 1.19 | 0.46 | -0.26 | -0.04 | 0.02 | 0.67 | -0.11 | 0.06 | -0.43 | 0.96 | -0.40 | 0.53 |

Appendix A (Continued)

|  | V15 | V16 | V17 | V18 | V19 | V20 | V21 | V22 | V23 | V24 | V25 | V26 | V27 | V28 |
| --- | --- | --- | --- | --- | --- | --- | --- | --- | --- | --- | --- | --- | --- | --- |
| Mean | 4.04 | 3.70 | 6.1 | 4.94 | 5.78 | 5.61 | 4.39 | 4.14 | 1.73 | 3.53 | 5.62 | 4.07 | 3.06 | 3.83 |
| SD | 2.11 | 2.25 | 1.75 | 1.93 | 1.80 | 1.86 | 2.06 | 2.21 | 1.39 | 2.13 | 2.02 | 2.23 | 1.94 | 2.29 |
| Kurtosis | -0.96 | -1.12 | 0.19 | -0.79 | -0.22 | -0.44 | -1.05 | -1.21 | 4.48 | -0.92 | -0.5 | -1.08 | -0.25 | -1.18 |
| Skewness | 0.33 | 0.37 | -0.86 | -0.17 | -0.71 | -0.58 | -0.04 | 0.04 | 2.20 | 0.45 | -0.67 | 0.21 | 0.8 | 0.31 |
| V1 | .05 | .05 | .09 | .07 | .12 | .22 | .14 | .12 | .14 | .17 | .12 | .15 | .18 | .14 |
| V2 | .11 | .21 | .03 | .20 | .06 | .12 | .16 | .25 | .24 | .16 | .09 | .15 | .20 | .11 |
| V3 | .09 | .22 | .04 | .14 | .08 | .15 | .22 | .13 | .25 | .19 | .09 | .23 | .26 | .14 |
| V4 | .17 | .24 | .26 | .28 | .24 | .26 | .23 | .19 | .11 | .14 | .24 | .28 | .30 | .20 |
| V5 | .11 | .24 | .24 | .27 | .27 | .24 | .20 | .13 | .06 | .13 | .19 | .26 | .25 | .16 |
| V6 | .20 | .29 | .24 | .35 | .20 | .23 | .26 | .22 | .09 | .14 | .24 | .24 | .23 | .23 |
| V7 | .14 | .28 | .18 | .24 | .24 | .21 | .29 | .15 | .17 | .23 | .21 | .23 | .27 | .14 |
| V8 | .12 | .29 | .20 | .32 | .31 | .29 | .29 | .32 | .27 | .26 | .23 | .36 | .38 | .25 |
| V9 | .18 | .21 | .42 | .38 | .29 | .27 | .15 | .22 | .06 | .18 | .27 | .26 | .23 | .17 |
| V10 | .04 | .29 | .16 | .22 | .22 | .22 | .21 | .24 | .14 | .19 | .12 | .18 | .21 | .16 |
| V11 | .16 | .33 | .43 | .39 | .30 | .23 | .26 | .21 | .08 | .14 | .27 | .21 | .21 | .14 |
| V12 | .21 | .33 | .19 | .36 | .23 | .22 | .28 | .26 | .33 | .24 | .18 | .30 | .35 | .22 |
| V13 | .15 | .30 | .40 | .25 | .31 | .29 | .19 | .13 | .03 | .12 | .35 | .25 | .18 | .20 |
| V14 | .12 | .25 | .20 | .19 | .12 | .11 | .28 | .21 | .18 | .10 | .23 | .19 | .21 | .13 |
| V15 | 1 | .25 | .22 | .17 | .18 | .03 | .09 | .03 | .12 | .07 | .07 | .11 | .07 | .06 |
| V16 | .22 | 1 | .27 | .28 | .30 | .22 | .27 | .25 | .23 | .16 | .23 | .33 | .29 | .33 |
| V17 | .25 | .25 | 1 | .45 | .52 | .42 | .25 | .22 | -.04 | .11 | .45 | .25 | .15 | .12 |
| V18 | .13 | .25 | .43 | 1 | .45 | .45 | .35 | .35 | .12 | .21 | .31 | .41 | .31 | .25 |
| V19 | .28 | .23 | .49 | .40 | 1 | .50 | .30 | .30 | .01 | .21 | .41 | .31 | .25 | .21 |
| V20 | .04 | .14 | .37 | .41 | .42 | 1 | .38 | .39 | .09 | .31 | .30 | .32 | .33 | .22 |
| V21 | .18 | .22 | .2 | .36 | .27 | .39 | 1 | .48 | .20 | .46 | .27 | .31 | .35 | .23 |
| V22 | .05 | .21 | .19 | .32 | .35 | .40 | .51 | 1 | .21 | .36 | .22 | .30 | .35 | .25 |
| V23 | .14 | .24 | -.03 | .10 | .04 | .11 | .15 | .18 | 1 | .27 | -.01 | .17 | .31 | .20 |
| V24 | .19 | .12 | .05 | .21 | .25 | .28 | .45 | .35 | .25 | 1 | .23 | .28 | .33 | .29 |
| V25 | .13 | .16 | .46 | .31 | .43 | .28 | .26 | .23 | .04 | .25 | 1 | .40 | .25 | .25 |
| V26 | .10 | .23 | .22 | .40 | .33 | .30 | .32 | .33 | .20 | .29 | .39 | 1 | .49 | .40 |
| V27 | .17 | .31 | .14 | .32 | .24 | .30 | .44 | .39 | .35 | .37 | .23 | .49 | 1 | .43 |
| V28 | .09 | .24 | .09 | .31 | .19 | .26 | .25 | .24 | .24 | .35 | .22 | .44 | .46 | 1 |
| Mean | 4.12 | 3.52 | 5.91 | 4.88 | 5.60 | 5.47 | 4.35 | 4.24 | 1.81 | 3.49 | 5.41 | 3.91 | 2.92 | 3.74 |
| SD | 2.13 | 2.16 | 1.85 | 1.85 | 1.87 | 1.80 | 2.01 | 2.21 | 1.49 | 2.11 | 2.06 | 2.21 | 1.90 | 2.30 |
| Kurtosis | -0.99 | -0.94 | -0.43 | -0.86 | -0.7 | -0.47 | -0.97 | -1.22 | 3.96 | -0.87 | -0.72 | -1.06 | 0.02 | -1.17 |
| Skewness | 0.28 | 0.49 | -0.65 | -0.01 | -0.52 | -0.51 | -0.04 | -0.06 | 2.12 | 0.49 | -0.53 | 0.25 | 0.92 | 0.34 |

*Note.* All items were measured on an 8-point Likert scale.
